# Adjuvant gemcitabine versus NEOadjuvant gemcitabine/oxaliplatin plus adjuvant gemcitabine in resectable pancreatic cancer: a randomized multicenter phase III study (NEOPAC study)

**DOI:** 10.1186/1471-2407-11-346

**Published:** 2011-08-10

**Authors:** Stefan Heinrich, Bernhard Pestalozzi, Mickael Lesurtel, Frederik Berrevoet, Stéphanie Laurent, Jean-Robert Delpero, Jean-Luc Raoul, Phillippe Bachellier, Patrick Dufour, Markus Moehler, Achim Weber, Hauke Lang, Xavier Rogiers, Pierre-Alain Clavien

**Affiliations:** 1 Department of General and Abdominal Surgery, University Hospital of Mainz, Langenbeck-strasse 1, 55118 Mainz, Germany; 2 Department of Medical Oncology, University Hospital of Zurich, Raemistrasse 100, 8091 Zuerich, Switzerland; 3 Visceral and Transplantation Surgery, University Hospital of Zurich, Raemistrasse 100, 8091 Zuerich, Switzerland; 4 Department of General and Hepatobiliary Surgery University Hospital of Ghent, De Pintelaan 185, B-9000 Ghent, Belgium; 5 Department of Gastroenterology, Digestive Oncology, University Hospital of Ghent, De Pintelaan 185, B-9000 Ghent, Belgium; 6 Department of Surgery, Institut Pauli Calmettes, 232 Bd Ste Marguerite, 13273 Marseille, France; 7 Department of Oncology, Institut Pauli Calmettes, 232 Bd Ste Marguerite, 13273 Marseille, France; 8 Department of Visceral Surgery, University Hospital of Strasbourg, Hôpital Hautepierre, Avenue Molière, 67098 Strasbourg, France; 9 Department of Oncology, University Hospital of Strasbourg, Hôpital Hautepierre, Avenue Molière, 67098 Strasbourg, France; 10 Department of Internal Medicine, Gastroenterology/Gastrointestinal Oncology, University Hospital of Mainz, Langenbeckstrasse 1, 55118 Mainz, Germany; 11 Department of Pathology, University Hospital of Zurich, Raemistrasse 100, 8091 Zuerich, Switzerland

## Abstract

**Background:**

Despite major improvements in the perioperative outcome of pancreas surgery, the prognosis of pancreatic cancer after curative resection remains poor. Adjuvant chemotherapy increases disease-free and overall survival, but this treatment cannot be offered to a significant proportion of patients due to the surgical morbidity. In contrast, almost all patients can receive (neo)adjuvant chemotherapy before surgery. This treatment is safe and effective, and has resulted in a median survival of 26.5 months in a recent phase II trial. Moreover, neoadjuvant chemotherapy improves the nutritional status of patients with pancreatic cancer. This multicenter phase III trial (NEOPAC) has been designed to explore the efficacy of neoadjuvant chemotherapy.

**Methods/Design:**

This is a prospective randomized phase III trial. Patients with resectable cytologically proven adenocarcinoma of the pancreatic head are eligible for this study. All patients must be at least 18 years old and must provide written informed consent. An infiltration of the superior mesenteric vein > 180° or major visceral arteries are considered exclusion criteria. Eligible patients will be randomized to surgery followed by adjuvant gemcitabine (1000 mg/m^2^) for 6 months or neoadjuvant chemotherapy (gemcitabine 1000 mg/m^2^, oxaliplatin 100 mg/m^2^) followed by surgery and the same adjuvant treatment. Neoadjuvant chemotherapy is given four times every two weeks. The staging as well as the restaging protocol after neoadjuvant chemotherapy include computed tomography of chest and abdomen and diagnostic laparoscopy. The primary study endpoint is progression-free survival. According to the sample size calculation, 155 patients need to be randomized to each treatment arm. Disease recurrence will be documented by scheduled computed tomography scans 9, 12, 15, 21 and thereafter every 6 months until disease progression. For quality control, circumferential resection margins are marked intraoperatively, and representative histological sections will be centrally reviewed by a dedicated pathologist.

**Discussion:**

The NEOPAC study will determine the efficacy of neoadjuvant chemotherapy in pancreatic cancer for the first time and offers a unique potential for translational research. Furthermore, this trial will provide the unbiased overall survival of all patients undergoing surgery for resectable cancer of the pancreatic head.

**Trial registration:**

clinicalTrials.gov NCT01314027

## Background

Mortality rates of pancreatic surgery have dramatically decreased to less than 2% in experienced centers during the last decades. Despite these improvements in the perioperative outcome, long-term survival of patients with pancreatic cancer remains limited with only 12 months median survival reported from pure surgical series [1].

Similar to other gastrointestinal cancers, several adjuvant treatment concepts have been tested in the past to improve long-term outcome. After controversial results from initial chemoradiation trials [2,3] recent randomized trials demonstrate a significant prolongation of disease-free and overall survival by adjuvant chemotherapy [4-6]. While gemcitabine (Gem) and 5-fluorouracil(FU)/folinic acid (FA) are equally effective, adjuvant Gem is less toxic than 5-FU/FA [7]. Therefore, adjuvant chemotherapy with Gem should presently be considered the standard of care after a curative resection of pancreatic cancer. The major disadvantage of adjuvant chemotherapy is, however, that a large proportion of patients (> 20%) cannot receive any treatment [2,8,9], mainly due to the surgical morbidity of pancreatic surgery [10].

In contrast, a preoperative (neoadjuvant) treatment can be applied to almost all patients since it is independent of the surgical morbidity. The safety of this concept has been demonstrated in a recent phase II trial: 28 patients with resectable cancer of the pancreatic head received gemcitabine and cisplatin (GemCis) for two months before resection. This treatment was well tolerated with only a few grade III toxicities and al low surgical morbidity rate [11]. Furthermore, a significant histological and cytological response was documented resulting in a median survival of 26.5 months [12]. Interestingly, more than 40% of the patients were malnourished at study entry, and the nutritional status of these patients improved significantly during neoadjuvant chemotherapy [13]. In addition, neoadjuvant chemotherapy may decrease the amount of circulating tumor cells and intraoperative tumor spillage as demonstrated for several tumor entities [14].

Similar survival results have been reported from a recent phase II trial, in which patients with locally unresectable pancreatic cancer received a down-sizing chemotherapy with Gem and oxaliplatin (Ox): fourty percent of these patients finally underwent resection, and the R0 resection rate was 70% [15].

Following these encouraging results, this randomized phase III study was initiated to further investigate the efficacy of neoadjuvant chemotherapy for resectable cancer of the pancreatic head.

## Methods/Design

This is a randomized controlled phase III trial organized by the Swiss HPB-center at the University Hospital of Zurich. The study protocol has been approved by the Ethical Review Board of the University Hospital of Zurich and the Swiss national authorities. Each approved country has a national main investigator for the coordination of the national centers.

**Figure 1:**
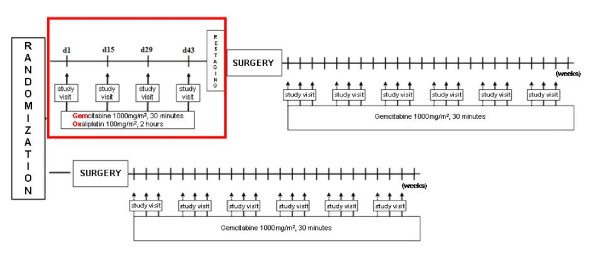
**Schematic description of the NEOPAC trial**. Patients are randomized to either receive the standard treatment (surgery+adjuvant chemotherapy) or neoadjuvant chemotherapy followed by the standard treatment.

Eligible patients are randomized (1:1) to either receive the standard treatment (pancreaticoduodenectomy and adjuvant chemotherapy) or neoadjuvant chemotherapy followed by this standard treatment (Figure 1).

### Eligibility criteria

Patients (> 18 years) with resectable cytologically proven adenocarcinoma of the pancreatic head without general contraindications for a pancreatico-duodenectomy are eligible for this study (table 1). All patients have to provide written informed consent.

**Table 1:** Eligibility criteria for the NEOPAC trial.

Inclusion criteria
resectable adenocarcinoma of the pancreatic head
- T1-3, Nx, M0 (UICC 6^th ^version, 2002)
cytologic or histologic confirmation of adenocarcinoma age > 18 years
written informed consent

Exclusion criteria
contraindication for duodenopancreatectomy
distant metastases
infiltration > 180° of the portal vein
abutment of the tumor to the SMA infiltration of the SMA or the celiac trunk
chronic neuropathy > grade 2
WHO performance score > 2
uncorrectable cholestasis (bilirubin > 100 mmol/l despite drainage attempts for more than four weeks prior to inclusion)
female patients in child bearing age not using adequate contraception
pregnant or lactating women
mental or organic disorders which could interfere with informed consent or treatments
Second malignancy within the past 5 years, except non-melanomatous skin or non-invasive cervical cancer
percutaneous tumor biopsy

**Figure 2:**
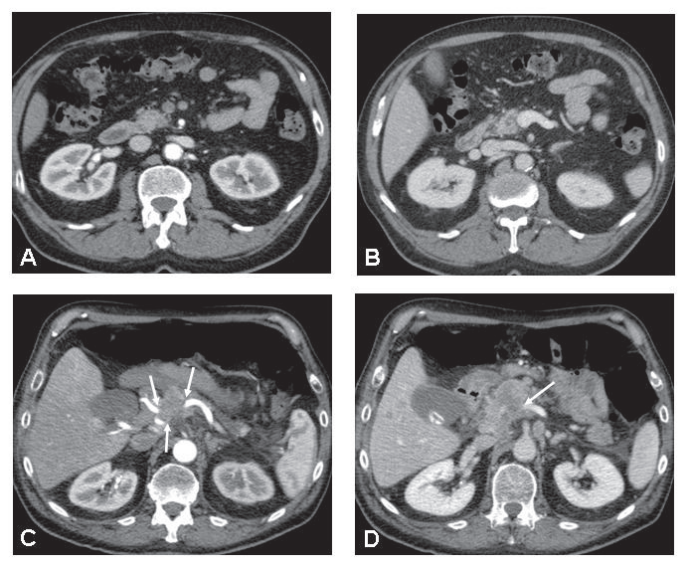
**Contrast-enhanced CT (ceCT) shows a tumor-free superior mesenteric artery (sma) [A] and portal vein [B]**. In another patient, ceCT suggests tumor infiltration of the celiac trunk [C] and the portal vein confluence [D] (arrows).

Tumors with portal vein infiltration of more than 180°, tumor abutment of major visceral arteries (T4) or distant metastases (M1) are excluded from this study (Figure 2). A previous percutaneous biopsy of the primary tumor is considered an exclusion criterion as well as a chronic neuropathy or WHO performance status > °2. Also, women of childbearing age not using adequate contraception as well as pregnant and lactating women are excluded. Furthermore, mental or organic disorders which interfere with the informed consent or the treatment are considered exclusion criteria. Finally, a second malignancy diagnosed within the past 5 years, except for non-melanomatous skin cancer or non-invasive cervical cancer.

### Staging examinations

Before study inclusion, distant metastases are ruled out by contrast-enhanced (ce)CT scan of chest and abdomen. Furthermore, all patients require a diagnostic laparoscopy to exclude peritoneal or small hepatic metastases. If positron-emission tomography (PET)/CT is available, centers may decide to use PET/CT for staging and restaging in all patients. Local resectability is also determined by ceCT. Fine needle aspiration cytology (FNA) by endoscopic ultrasound or brush cytology by endoscopic retrograde cholangio-pancreatography (ERCP) is required to confirm pancreatic cancer prior to study inclusion.

ceCT (or PET/CT) as well as diagnostic laparoscopy are repeated after neoadjuvant chemotherapy in order to exclude unresectability due to disease progression.

### Chemotherapy

Neoadjuvant chemotherapy consists of 4 bi-weekly cycles of gemcitabine (1000 mg/m^2^) and oxaliplatin (100 mg/m^2^).

After pancreas resection, all patients receive, adjuvant chemotherapy with 6 cycles of gemcitabine 1000 mg/m^2 ^(d1, d8, d15). Side-effects of chemotherapy are graded by the "Common Terminology Criteria for Adverse Events" version 3 http://ctep.cancer.gov/forms/CTCAEv3.pdf. At each study visit, laboratory parameters are determined for dose adjustments.

### Surgery

Surgery must be performed within 10-20 days after the last neoadjuvant GemOx infusion or as soon as possible in the control arm. Resection of the pancreatic head will be preferentially performed as a standard pancreaticoduodenectomy. Alternatively, pylorus-preserving resections or other reconstructions can be performed.

Surgical morbidity is assessed by the Dindo/Clavien classification [16]. Pancreatic fistula are defined by the definition of the international study group for pancreatic surgery (ISGPS) [17].

### Follow-up

The follow-up is based on physical examination, CA 19-9 measurement and CT scans of chest and abdomen 9, 12, 15 months after study inclusion and every six months thereafter until disease recurrence.

### Disease recurrence

Any newly appearing lesion with histological documentation of cancer defines recurrent disease. Also, any newly appearing lesion(s) suspicious for malignancy without histological documentation but increasing in size upon repeated follow-up exams especially in the context of progressive symptoms (pain, weight loss) or increasing tumor marker (CA 19-9) levels are considered metastases.

Also, any newly appearing or progressive soft tissue lesion(s) in the former bed of the pancreatic head suspicious for malignancy with either histologic documentation of cancer or increasing size on follow-up exams is considered (local) recurrence.

### Aim of the study

The aim of this study is to test the additional efficacy of neoadjuvant chemotherapy to the standard treatment for resectable cancer of the pancreatic head (surgery + adjuvant chemotherapy).

### Study endpoints

The primary study endpoint is the progression-free survival, which is defined by the date from written informed consent until disease progression or tumor recurrence (local or distant). E.g. the detection of peritoneal metastases during laparoscopy as well as unresectability during surgical exploration are considered disease progression.

Secondary end-points are the histological response to the neoadjuvant treatment, overall survival, complication rates after surgery, and feasibility of adjuvant chemotherapy.

### Sample-size calculation

Assuming a progression-free survival at one year after randomization of 55% for the neoadjuvant + standard treatment and 40% for the standard treatment, and an equal number of patients in each treatment arm (50/50), a total of 310 patients (155 per arm) are required for this two-arm trial to detect a significant difference with an alpha-error of 0.05 and a 1-β power value of 0.80. Interim analyses are scheduled after inclusion of 100 and 200 patients, and an independent data review board will decide upon continuation of the study.

### Quality control

This study will be performed in compliance with the study protocol, good clinical practice (GCP) and the applicable regulatory requirements.

Patient randomization and data collection are performed on a central database (secuTrial™), which allows continuous centralized data monitoring of all participating centers. Furthermore, scheduled audits will be performed at each study center by the coordinating center in Zurich.

**Figure 3:**
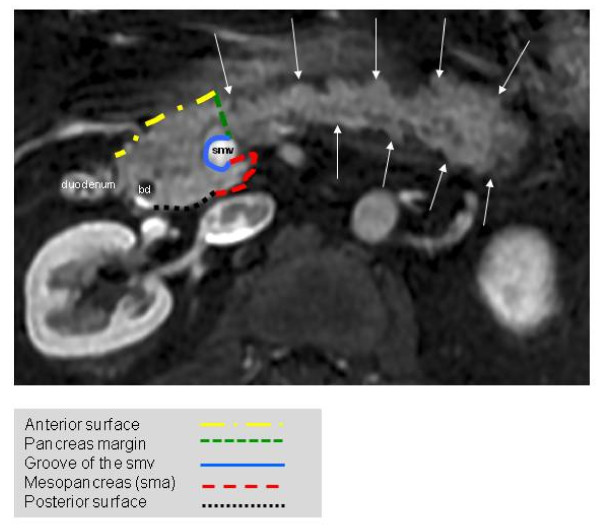
**Magnetic resonance imaging of the pancreas indicating the circumferential resection margins (bd: bile duct, smv: superior mesenteric vein, arrows indicate pancreatic tail)**.

Circumferential margins of the resected specimen are stained intraoperatively: dorsal, ventral, mesopancreatic margins as well as the mesenteric groove are stained separately (Figure 3). This will allow exact and reproducible assessment of R0 and R1 resections. Furthermore, a central pathological review will be performed by a dedicated pathologist to confirm the diagnosis of ductal adenocarcinoma (A.W.).

### Translational research

The quality of life (QoL) will be assessed by the QLQ-30 of the European Organization for Research and Treatment of Cancer (EORTC) at study inclusion, before (neoadjuvant arm) and after surgery, as well as at any follow-up visit. In addition, the histological response of the tumor to the neoadjuvant treatment will be assessed by established scores [11]. Tumor tissue will be stored to enable further translation research and assess potential predictive factors.

## Discussion

Adjuvant chemotherapy is considered a standard treatment after a curative resection of pancreatic cancer in many centers due to the improvements in disease-free and overall survival. However, a significant proportion of patients does not receive this treatment postoperatively due to the morbidity of pancreas surgery.

Neoadjuvant treatments are established concepts for rectal and gastric cancer [18,19], where the toxicity is better tolerated [19]. Furthermore, neoadjuvant treatments can be applied to almost all patients independent of the surgical morbidity, and helps improving the nutritional status in malnourished pancreatic cancer patients [11].

Very recently, the FOLFIRINOX regimen has been reported to be superior to a Gem-mono treatment in the palliative treatment of pancreatic cancer [20]. This treatment achieved a higher objective response rate (31.6% versus 9.4%), less quality of life impairment (31% versus 66%) and a better median progression-free survival (6.4 versus 3.3 months). However, FOLFIRINOX is more toxic than Gem-mono with grade III/IV neutropenia and diarrhea of 45% versus 21% and 12.7% versus 1.8%, respectively [20]. In contrast, the combination of gemcitabine and platin derivates is also more effective than Gem-mono treatment, but has minimal side-effects [21]. Therefore, the safety of this combination as neoadjuvant chemotherapy has been evaluated in a phase II-trial. In this trial, neoadjuvant treatment was safe and well tolerated [13].

The objective of the NEOPAC study is to determine for the first time the efficacy of this short-term neoadjuvant chemotherapy in addition to the current standard treatment. Based on the results of a phase II-trial, we hypothesize that the postoperative course of the pancreaticoduodenectomy will be less complicated following neoadjuvant chemotherapy due to the improvement of the nutritional status [13]. Therefore, these patients will presumably receive adjuvant chemotherapy to a higher percentage, which should result in a better long-term survival.

Since the NEOPAC trial tests neoadjuvant chemotherapy in primarily resectable disease, and not a down-sizing protocol, local resectability criteria are very strict, and the staging and re-staging protocols are extensive.

Furthermore, this study will reveal the percentage of patients eligible for adjuvant chemotherapy after surgery and provide the median survival of the entire cohort of patients with resectable cancer of the pancreatic head. In addition to these clinical questions, this study design offers a unique potential for translational research projects.

This study is a multicenter study, and recruitment has started. Any center with appropriate case load and logistics (e.g. EUS) interested in participating in this study is welcome to contact the principle investigator (PAC) for further information.

## List of abbreviations

Bd: Bile duct; ceCT: contrast-enhanced computed tomography; Cis: Cisplatin; CONKO: Charité Onkologie; ERCP: endoscopic retrograde cholangio-pancretaography; ESPAC: European study group for pancreatic cancer; EUS: endoscopic ultrasound; FNA: fine-needle aspiration; PET/CT: positron-emission-tomography/computed tomography; Gem: Gemcitabine; Ox: Oxaliplatin; QoL: Quality of life; QLQ-30: Quality-of-life-questionnaire 30; SMA: superior mesenteric artery; SMV: superior mesenteric vein.

## Competing interests

The authors declare that they have no competing interests.

## Authors' contributions

SH and BP designed the study and participate in the performance and coordination of the trial. ML, PB, KG, JRD, JLR, PB, PD, MM, HL and XR participate in the trial performance and patient recruitment. AW is the reference pathologist for confirmation of histological diagnosis and assessment of histological response, and PAC is the principle investigator. All authors have read and approved the final manuscript.

## Pre-publication history

The pre-publication history for this paper can be accessed here:

http://www.biomedcentral.com/1471-2407/11/346/prepub
